# ERVW-1 Activates ATF6-Mediated Unfolded Protein Response by Decreasing GANAB in Recent-Onset Schizophrenia

**DOI:** 10.3390/v15061298

**Published:** 2023-05-31

**Authors:** Xing Xue, Xiulin Wu, Lijuan Liu, Lianzhong Liu, Fan Zhu

**Affiliations:** 1 State Key Laboratory of Virology, Department of Medical Microbiology, School of Basic Medical Sciences, Wuhan University, Wuhan 430071, China; 2019203010007@whu.edu.cn (X.X.); 2017203010009@whu.edu.cn (X.W.); lijuanliu@whu.edu.cn (L.L.); 2 Hubei Province Key Laboratory of Allergy & Immunology, Wuhan University, Wuhan 430071, China; 3 Wuhan Mental Health Center, Wuhan 430071, China; liulianzhong@hotmail.com

**Keywords:** ERVW-1, GANAB, ATF6, XBP1, ER stress, unfolded protein response, schizophrenia

## Abstract

Schizophrenia, a mental disorder, afflicts 1% of the worldwide population. The dysregulation of homeostasis in the endoplasmic reticulum (ER) has been implicated in schizophrenia. Moreover, recent studies indicate that ER stress and the unfolded protein response (UPR) are linked to this mental disorder. Our previous research has verified that endogenous retrovirus group W member 1 envelope (ERVW-1), a risk factor for schizophrenia, is elevated in individuals with schizophrenia. Nevertheless, no literature is available regarding the underlying relationship between ER stress and ERVW-1 in schizophrenia. The aim of our research was to investigate the molecular mechanism connecting ER stress and ERVW-1 in schizophrenia. Here, we employed Gene Differential Expression Analysis to predict differentially expressed genes (DEGs) in the human prefrontal cortex of schizophrenic patients and identified aberrant expression of UPR-related genes. Subsequent research indicated that the UPR gene called *XBP1* had a positive correlation with *ATF6*, *BCL-2,* and *ERVW-1* in individuals with schizophrenia using Spearman correlation analysis. Furthermore, results from the enzyme-linked immunosorbent assay (ELISA) suggested increased serum protein levels of ATF6 and XBP1 in schizophrenic patients compared with healthy controls, exhibiting a strong correlation with ERVW-1 using median analysis and Mann–Whitney U analysis. However, serum GANAB levels were decreased in schizophrenic patients compared with controls and showed a significant negative correlation with ERVW-1, ATF6, and XBP1 in schizophrenic patients. Interestingly, in vitro experiments verified that ERVW-1 indeed increased ATF6 and XBP1 expression while decreasing GANAB expression. Additionally, the confocal microscope experiment suggested that ERVW-1 could impact the shape of the ER, leading to ER stress. GANAB was found to participate in ER stress regulated by ERVW-1. In conclusion, ERVW-1 induced ER stress by suppressing GANAB expression, thereby upregulating the expression of ATF6 and XBP1 and ultimately contributing to the development of schizophrenia.

## 1. Introduction

Schizophrenia is a debilitating mental disorder that affects approximately 0.5–1% of the worldwide population and has a significant economic impact, consuming 0.02–1.65% of the global gross product [1,2]. Schizophrenia is characterized by clinical cognitive impairments and is divided into three symptom categories: positive, negative, and cognitive symptoms [3]. Interestingly, endoplasmic reticulum (ER) stress has been found to interfere with neuroplasticity [4] and is closely associated with metabolic disorders in individuals with schizophrenia [5,6]. It has been reported that ER stress can cause brain impairment, leading to neuroinflammation and subsequent neuronal cell death in schizophrenia [7]. 

The ER plays a crucial role in protein synthesis, modification (e.g., N-linked glycosylation), and maintaining neuronal health and function via proteostasis under normal physiological conditions [8]. However, the accumulation of misfolded proteins and imbalance in protein homeostasis can lead to ER stress, triggering the unfolded protein response (UPR). The UPR consists of three major pathways: inositol requiring enzyme 1 (IRE1), activating transcription factor 6 (ATF6), and PKR-like ER kinase (PERK). IRE1 is a key pathway involved in ER stress. Its ribonuclease activity leads to the splicing of an intron from the inactive form of *X box binding protein 1 (XBP1)* mRNA, resulting in the production of *spliced XBP1 (sXBP1 or XBP1s)*. Spliced XBP1 becomes an active transcription factor that promotes protein degradation and folding [9]. *XBP1* splicing is considered an important marker of ER stress and has been implicated in the pathogenesis of schizophrenia [10,11]. A recent study by Kim et al. discovered increased levels of XBP1 protein and *spliced XBP1* mRNA in the dorsolateral prefrontal cortex of elderly individuals with schizophrenia [12]. The second UPR pathway involves ATF6, a leucine zipper protein encoded by *ATF6A* for ATF6 alpha and *ATF6B* for ATF6 beta. ATF6 is activated within the ER by misfolded proteins and transported to the Golgi apparatus, where it undergoes cleavage by a protease, resulting in the removal of approximately 400 amino acids from its N-terminal region. Activated ATF6 promotes the upregulation of UPR gene expression, including *XBP1* and *CHOP,* in the nucleus. ATF6 acts as both a sensor and an effector of ER stress [13]. The third pathway relies on the phosphorylation of protein kinase R (PKR)-like ER kinase (PERK). Phosphorylated PERK phosphorylates elongation initiation factor-2α (eIF2α) [14]. Interestingly, eIF2α phosphorylation reduces global protein translation while enhancing the translation of ATF6 [15]. The three major UPR pathways are directly or indirectly linked to ATF6, with XBP1, a risk factor for schizophrenia, being a downstream target gene of ATF6. N-glycosylation is essential to ER proteostasis, as it aids in glycoprotein folding, sorting, degradation, and secretion, ensuring proper protein folding [16]. Neutral alpha-glucosidase AB (GANAB), the catalytic subunit of glucosidase II, participates in N-glycosylation by specifically removing glucose residues from oligosaccharide precursors of immature glycoproteins [17]. N-glycosylation is associated with ER stress, as the dysregulation of protein glycosylation can induce ER stress and activate the UPR [18,19]. Moreover, GANAB, which mediates ATF6 transcriptional activation, has been identified as a novel prognostic marker of urothelial carcinoma [20]. However, the relationship among GANAB, ATF6, and schizophrenia has not been reported, and there is insufficient evidence to establish a correlation between UPR and schizophrenia. Even the pathogenesis of schizophrenia remains unclear.

Interestingly, multiple studies have reported an association between human endogenous retroviruses (HERVs) and schizophrenia [21,22,23]. HERVs are remnants of ancient germ-line cell infections by exogenous retroviruses and make up approximately 8% of the human genome [24]. HERVs share a similar internal structure with exogenous retroviruses, including gag (structural proteins), pol (enzymes), and env (envelope proteins), as well as 5′- and 3′-terminal long terminal repeats (LTRs). HERVs are classified into three groups: class I (gamma-retroviruses), class II (beta-retroviruses), and class III (spuma-retroviruses) [24,25]. Most HERVs are replication defective due to accumulated mutations and deletions. *Human endogenous retrovirus group W member 1 envelope* (*ERVW-1*; also named *HERV-W env*, *HERVWE1*, and *ERVWE1*), located on chromosome 7q21.2, belongs to class I and encodes a fusogenic protein called Syncytin-1 [26]. ERVW-1 consists of a surface subunit (SU) that mediates receptor recognition and a transmembrane subunit (TM) that acts as a class I viral fusion protein [27,28]. ERVW-1 is predominantly expressed in placenta cells, where it plays a role in fusion during pregnancy and placenta formation [29,30]. Various ecological factors, including pharmacological risk factors (e.g., caffeine and aspirin) [31] and viral risk factors (e.g., HBV X protein) [32], can influence ERVW-1 expression. Importantly, the abnormal expression of ERVW-1 has been implicated in various diseases, such as hepatocellular carcinoma [33], urothelial cell carcinoma [34], multiple sclerosis (MS) [35], and schizophrenia [36]. Furthermore, ERVW-1 has been detected in both cerebrospinal fluids (CSFs) and serum of schizophrenic patients [37,38]. Our previous studies have shown a significant increase in ERVW-1 levels in the blood of recent-onset schizophrenic patients [36,39,40,41,42,43,44]. However, to the best of our knowledge, no literature has reported the relationship among ERVW-1, UPR, and schizophrenia.

In this study, we investigated the differential expression of UPR-related genes in the prefrontal cortex of individuals with schizophrenia and healthy controls using bioinformatic analysis. Our findings reveal a positive correlation between the UPR gene *XBP1* and *ERVW-1* in schizophrenic patients. Additionally, our clinical results showed significantly increased levels of ATF6 and XBP1 proteins in the serum of schizophrenic patients compared with healthy controls, while the expression of GANAB was decreased. Furthermore, GANAB, ATF6, and XBP1 exhibited a strong correlation with ERVW-1 in schizophrenic patients. Intriguingly, cytological experiments demonstrated that ERVW-1 enhanced XBP1 expression and splicing by upregulating ATF6, which was counteracted by GANAB in SH-SY5Y cells. Moreover, we observed that ERVW-1 influenced ER morphology, and GANAB played a role in modulating these ER morphology changes induced by ERVW-1. Overall, our study provides novel insights into the molecular mechanisms associated with schizophrenia. 

## 2. Materials and Methods

### 2.1. Gene Differential Expression Analysis

We obtained the RNA microarray dataset (GSE12649) [45] on the human prefrontal cortex (BA46) of individuals with schizophrenia and healthy controls using the GPL96 platform (Affymetrix Human Genome U133A Array). The dataset was downloaded from Gene Expression Omnibus (GEO) using the R programming language called GEO query. To annotate hybridization signals and acquire gene expression information, we utilized the Bioconductor package hug133a.db. Differential expression analysis was conducted between individuals with schizophrenia and healthy controls using the Limma package in R. A significance threshold of *p* < 0.05 was applied for all statistical analyses, which were performed using the R programming language. The Sanger box (http://sangerbox.com/), accessed on 27 September 2022, was used to generate volcano plots and heat maps to visualize the differentially expressed genes (DEGs) [46]. Additionally, DNA hybridization array data from the GSE21138 dataset on the GPL570 platform (Affymetrix Human Genome U133 Plus 2.0 Array) [47] was obtained using the GEO query package. Hybridization signals were annotated, and gene expression was extracted using the Bioconductor package hug133plus2.db. The differential expression analysis of the gene expression data was performed using the R package Limma.

### 2.2. Gene Set Enrichment Analysis (GSEA) and Overlap Analysis

To assess the impact of the differentially expressed genes (DEGs) from GSE12649 and GSE21138 on schizophrenia, we performed hypergeometric distribution analysis using the Sanger box. Gene Set Enrichment Analysis (GSEA) was conducted to evaluate the enrichment of KEGG pathways and Gene Ontology (GO) biological processes. Specifically, we analyzed the overlap between the DEGs from GSE21138 and the gene set “GOBP RESPONSE TO ENDOPLASMIC RETICULUM STRESS” from Molecular Signatures Database (MSigDB) using the Sanger box.

### 2.3. Correlation Analysis

We conducted a correlation matrix analysis to explore the relationship between ERVW-1 and ER stress-related genes (*XBP1*, *ATF6*, *DDIT3*, *ERN1*, *BCL2*, and *ATF6B*) in the GSE21138 dataset using the Spearman correlation coefficient. The schizophrenia expression profile data from GSE21138 were used to assess the Spearman correlation between ERVW-1 and XBP1, between ATF6 and XBP1, and between BCL2 and XBP1. The visual analysis of the correlations was performed using the ggstatsplot package in the R programming language. Correlations were considered significant if the *p*-value was less than 0.05 and the absolute value of the correlation coefficient (|R^2^|) was greater than 0.02.

### 2.4. Serum Samples

We collected blood samples from a total of 39 schizophrenic patients and 36 healthy individuals at Wuhan Mental Health Center. Detailed information can be found in Table S1. Psychiatric symptoms were evaluated according to *Diagnostic and Statistical Manual of Mental Disorders*, 5th edition. All schizophrenic patients met the criteria for schizophrenia-related psychoses and confirmed that they had not received any medication treatment prior to sample collection. Patients with inflammatory conditions, acute infections, or neurological diseases were excluded from the study. The healthy controls were also excluded if they had any psychiatric or neurological disease. Serum samples were obtained using centrifugation at 1000× *g* for 10 min and stored at −80 °C until further use.

### 2.5. ELISA

Serum samples were 2-fold diluted by adding an equal volume of diluent, resulting in a final volume of 50 μL. The protein levels of ERVW-1, GANAB, ATF6, and XBP1 were analyzed using commercial enzyme-linked immunosorbent assay (ELISA) kits (ERVW-1 ELISA Kit (NM-61410H1; MEIMIAN, Yancheng, China), GANAB ELISA Kit (NM-50627H1; MEIMIAN, Yancheng, China), ATF6 ELISA Kit (NM-14916H1; MEIMIAN, Yancheng, China), and XBP1 ELISA Kit (NM-62347H1; MEIMIAN, Yancheng, China)). The assays were performed following the manufacturer’s protocols. After incubation, absorbance at 450 nm was measured using an enzyme-labeled instrument (Thermo Multiskan™ FC; Waltham, MA, USA). Standard curves were plotted, and the protein levels of ERVW-1, GANAB, ATF6, and XBP1s were calculated according to the manufacturer’s protocols.

### 2.6. Plasmid and Plasmid Constructs

The plasmid pCMV-ERVW-1 [39] was obtained as previously described. Primer premier 5.0 was used to design the primers. Homo sapiens *GANAB* (NM 001278192) was amplified using primers P1(5′-GGAATTCCATGGCGGCGGTAGCGGCAGTG-3′) and P2(5′-CCCAAGCTTGGGTATC GCAGGTGAATACTCCAAT-3′). The amplified fragment was inserted into the pcDNA3.1 vector using NheI and AflIII (Invitrogen, Carlsbad, CA, USA). 

One short hairpin RNA (shRNA) targeting *ATF6* (5′-AAGACTGGGAGTCGACGTT GT-3′) and the control shRNA (5′-GGTCGCTGTTTGACATTAT-3′) were inserted into the pSilencer 2.1-U6 neo shRNA expression vector (AM5764; Ambion Inc., Austin, TX, USA) using BamHI and Hind III. The amplified primers were as follows: forward primer, 5′-GATCCAAGACTGGGAGTCGACGTTGTTTCAAGAGAACAACGTCGACTCCCAGTCTTTTTTTTGGAAA-3′; reverse primer, 5′- AGCTTTTCCAAAAAAA AGACTGGGAGTCGACGTTGTTCTCTTGAAACAACGTCGACTCCCAGTCTTG-3′. All constructs were confirmed using sequencing (Sangon Biotech, Shanghai, China).

### 2.7. Cell Culture and Transfection

The human neuroblastoma cell line SH-SY5Y was purchased from American Type Culture Collection (ATCC; Manassas, VA, USA). SH-SY5Y cells were maintained in a mixture of essential medium (2225320; Gibco, Gaithersburg, MD, USA) and F-12 nutrient mixture (2209586; Gibco) in a 1:1 ratio, supplemented with 100 mmol/L sodium pyruvate (Gibco, Waltham, MA, USA), 10% fetal bovine serum (2001003; Biological Industries, Beit HaEmek, Israel), and 100 U/mL penicillin–streptomycin (2211093; Gibco), at 37 °C with 5% CO_2_. Plasmids were transfected into the cells using Turbofect transfection reagent (Thermo, Waltham, MA, USA) following the manufacturer’s instructions. Cells were collected for further analyses 48 hours post-transfection.

### 2.8. RNA Extraction, Reverse Transcription, and Quantitative PCR

Total RNA was extracted from 1 × 10^6^ cells using TRIzol reagent (15596018; Invitrogen), followed by chloroform separation and isopropanol precipitation. To remove any genomic DNA contamination, the extracted RNA was treated with RNase-free DNase (Promega, Madison, WI, USA). Then, 1 μg of total RNA was used for cDNA synthesis using ReverTra Ace qPCR RT master Mix (FSQ-301; TOYOBO) according to the manufacturer’s instructions. Thermo Scientific Strips of 8 Tubes and SYBR Green qPCR Master Mix (Invitrogen) were used for real-time quantitative PCR. The relative mRNA levels were calculated using the 2^−ΔΔCt^ method, with GAPDH serving as an internal reference (ΔΔCt = ΔCt (a target sample) −ΔCt (a control sample), ΔCt = Ct (Target) − Ct (GAPDH)). The primers were designed using primer 5.0 based on NCBI sequences. All primers are listed in Table S2. The primers were diluted to a concentration of 3.3 μmol/L with double distilled water, and the PCR program was as follows: 95 °C for 3 min, followed by 40 cycles at 95 for 45 s, 56 °C for 45 s, and 72 °C for 45 s.

### 2.9. Protein Extraction and Western Blot

Cultured SH-SY5Y cells were lysed using M-PER TM mammalian protein extraction reagent (UC282138; Thermo Fisher Scientific, Waltham, MA, USA) supplemented with protease and phosphatase inhibitors (Roche Applied Science, Indianapolis, IN, USA). Protein separation was performed using 10% SDS-PAGE, and the proteins were subsequently electroblotted onto PVDF membranes (Millipore, Burlington, MA, USA). Before protein transfer, the membrane was inactivated by incubating with Milli-Q^®^ water for 1–2 min to completely replace methanol. To block the membrane and dilute primary antibodies, a 5% nonfat dry milk solution in TBST was prepared. The membranes were then incubated with the following primary antibodies: rabbit anti-ATF6 pAb (1: 1000; A0202; Abclonal, Wuhan, China), rabbit anti-GANAB (1: 1000; A19876; Abclonal, China), rabbit anti-XBP1 pAb (1: 1000; A1731; Abclonal, China), anti-ERVW-1 pAb (1: 1000; A16522; Abclonal, China), and anti-GAPDH (1:10,000; ab8245; Abcam, Waltham, MA, USA). The membranes were incubated overnight at 4 °C with primary antibodies, followed by incubation with secondary antibodies (HRP-conjugated anti-rabbit/mouse) at room temperature for 1 h. Protein expression was visualized using ECL kits (Millipore, USA) and analyzed using a chemiluminescence gel imaging system (Amersham, Uppsala, Sweden). The intensity of the Western blot bands was analyzed using Image J 1.8.0- Windows 8 software.

### 2.10. Confocal Microscopy

A total of 5 × 10^3^ cells were seeded in each confocal dish. Immunofluorescence signals were examined using a confocal laser scanning microscope (TCS SP8; Leica Microsystems, Wetzlar, Germany) with an HCX PLAPO 63*/1.40 oil objective lens. Plasmids pCMV/pCMV- ERVW-1 and pcDNA3.1-GANAB were transfected into SH-SY5Y cells to study the function and structure of the ER. To visualize the ER, cells were treated with 1 μmol/L ER-Tracker Red dye (1:1000; C1041; Beyotime Biotechnology, Shanghai, China) in medium and incubated for 15 min at 37 °C. Subsequently, the nucleus was stained with DAPI (1:200; C1005; Beyotime Biotechnology, China) for 15 min at 37 °C. The collected images were projected and analyzed in two-dimensional planes using red (ER; excitation spectrum: 587 nm) and blue (DAPI; spectrum excitation: 405 nm) displays.

### 2.11. Statistical Analysis

For the clinical results, we used median and nonparametric analyses. The relationships among ERVW-1, GANAB, ATF6, and XBP1 were analyzed using correlation analysis.

The data were presented as means ± SDs (standard deviations). All experiments were repeated three times. Statistical analyses were performed using GraphPad Prism 5 and IBM SPSS software Statistics 20. *p* < 0.05 was considered statistically significant.

## 3. Results

### 3.1. Differentially Expressed UPR Genes and ER Stress Enrichment in Schizophrenia

**Figure 1:**
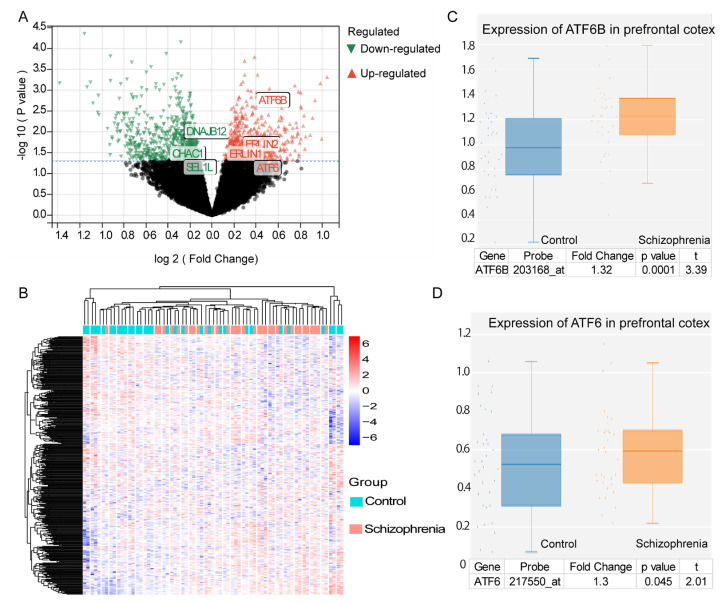

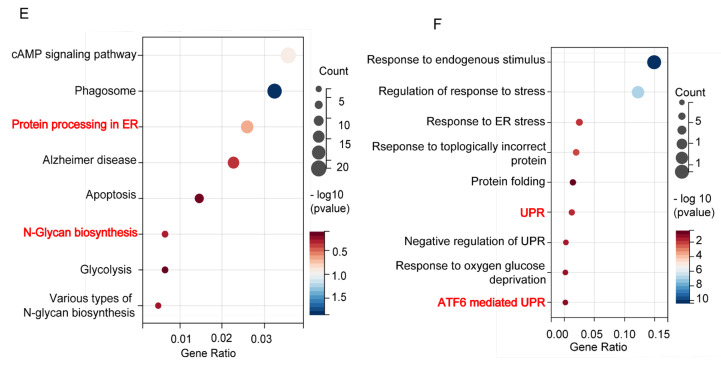
ER stress-related genes were significantly differential expressed in schizophrenic patients compared with healthy controls, and the DEGs of the schizophrenia dataset GSE12649 were enriched in ER stress and N-glycan-related pathways and biological processes. (**A**) Volcano plot shows the 640 upregulated genes containing ATF6 and ATF6B, and 667 downregulated genes. Black dots indicate no significantly differentially expressed genes; red dots indicate significantly differentially upregulated genes; and green dots indicate significantly downregulated genes; *p* < 0.05, |log_2_(Fold Change) |>1. (**B**) Heatmap of expression of DEGs. (**C**) Significantly increased expression of ATF6B in schizophrenia (*p* < 0.05, fold change = 1.32). (**D**) Significantly increased expression of ATF6 in schizophrenia (*p* < 0.05, fold change = 1.30). (**E**) KEGG enrichment analysis showed that the DEGs of GSE12649 were significantly enriched in protein process in the ER and N-glycan biosynthesis. (**F**) GO biological processes enrichment analysis showed that the DEGs of GSE12649 were significantly enriched in the ER UPR and ATF6-mediated UPR.

ER stress has an impressive connection with cellular homeostasis, suggesting its potential involvement in the pathogenesis of schizophrenia [48]. ATF4, an essential gene in the UPR triggered by ER stress, serves as a biomarker of schizophrenia [49]. To further understand the relationship between ER stress and schizophrenia, we analyzed GSE12649, a schizophrenia RNA microarray dataset from the human prefrontal cortex. Using the Limma package in the R program, we identified 640 upregulated genes and 667 downregulated genes (log2(FC)| > 1, *p*< 0.05). A volcano plot was generated to visualize the DEGs between schizophrenic patients and healthy individuals at the mRNA level (Figure 1A). Heat maps displaying the expression values of all DEGs in each sample were also created (Figure 1B). We identified 28 genes that were common to both the DEGs and the gene set called “GOBP RESPONSE TO ENDOPLASMIC RETICULUM STRESS” (Figure S1A,B). Among the upregulated genes, *ATF6B* was found to be increased by 1.32 times in the mRNA level of schizophrenic patients compared with normal controls (Figure 1C). *ATF6* and *ATF6B* are homologs [50], so it is not surprising that the ATF6 mRNA level was also increased by 1.3 times in individuals with schizophrenia compared with healthy controls (Figure 1D).

Subsequently, the results of KEGG pathway analysis revealed that these DEGs were enriched in several pathways, including protein process in the ER, cAMP signaling pathway, phagosome, Alzheimer’s disease, apoptosis, N-glycan biosynthesis, glycolysis, and various types of N-glycan biosynthesis (Figure 1E), suggesting that the DEGs were primarily enriched in proteostasis-related pathways, particularly protein processing in the ER. Furthermore, the enrichment analysis of GO biological processes suggested that the DEGs were involved in various biological processes, including UPR, ATF6-mediated UPR, negative regulation of UPR, protein folding, response to endogenous stimuli, regulation of response to stress, response to ER stress, response to topologically incorrect protein, and response to oxygen–glucose deprivation, indicating that the DEGs were mainly associated with biological processes related to proteases, as indicated by the UPR (Figure 1F). Similarly, the GO cellular component enrichment analysis of DEGs showed that they were predominantly located in endomembranous systems, particularly the ER (Figure S1C). Additionally, we observed enrichment of UPR-related molecular functions among the DEGs, including transcriptional regulator activity, sequence-specific DNA binding, DNA binding transcription factor activity, and DNA binding transcription activator activity (Figure S1D). In conclusion, those DEGs were mainly enriched in UPR-associated genes in schizophrenic patients.

### 3.2. ERVW-1 Correlated with ER Stress Indictor XBP1 in Schizophrenia

**Figure 2:**
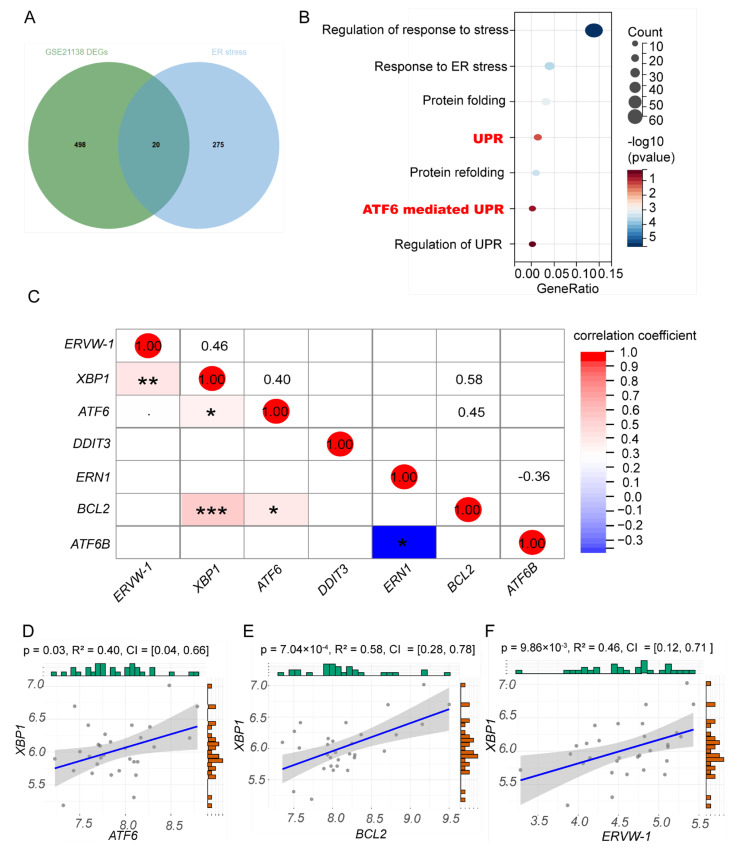
DEGs in GSE21138 were overlapped with and enriched in ER stress-related genes, and *XBP1* was significantly correlated with ERVW-1 in schizophrenia. (**A**) Overlap analysis showed 20 common genes between 517 DEGs and 295 ER stress genes. (**B**) GO biological processes enrichment analysis showed that DEGs in GSE21138 were enriched in the UPR. (**C**) Multiple correlation of ER stress-related genes based on ERVW-1 showed that XBP1 was significantly positively correlated with *ERVW-1*, *ATF6*, and *BCL-2*. (**D**) Linear regression analysis showed significant positive correlation of *ATF6* and *XBP1* in schizophrenia (*p* < 0.05; R^2^ = 0.40). (**E**) Significant positive correlation of *BCL2* and *XBP1* in schizophrenia (*p* < 0.001; R^2^ = 0.58). (**F**) Significant positive correlation of *XBP1* and *ERVW-1* in schizophrenia (*p* < 0.01; R^2^ = 0.45). * *p* < 0.05, ** *p* < 0.01, *** *p* < 0.001.

Previous studies have demonstrated increased transcription of ERVW-1 in schizophrenia [39,51]. Furthermore, the evidence from our lab proves that ERVW-1 may contribute to the development of schizophrenia via various mechanisms and that it is an important risk factor for schizophrenia [39,40,41,42,43,44,51,52,53,54,55,56,57]. Additionally, Li et al. have reported a significant increase in the transcriptional level of ERVW-1 in brain mRNA-seq data using publicly available RNA sequencing datasets [58]. Based on the results of the DEG screening in schizophrenia and our previous research, we identified the genes related to ERVW-1 and ER stress in schizophrenia. Among the 517 DEGs in GSE21138, we found 20 common genes between the DEGs and ER stress (Figure 2A). The enrichment analysis of GO biological processes revealed that the DEGs in GSE21138 were significantly enriched in the UPR (Figure 2B). In schizophrenic patients from GSE21138, we obtained the expression values of ERVW-1 and ATF6-mediated UPR genes, including *XBP1, ATF6, DDIT3,*

*ERN1,* and *BCL2*. We analyzed the correlations between ERVW-1 and the ATF6-mediated UPR-related genes and visualized them using a heatmap. We found a significant positive correlation between *XBP1*, and *ATF6*, *BCL-2*, and *ERVW-1* (Figure 2C). However, there was a significant negative correlation between *ATF6B* and *ERN1* (Figure 2C). Furthermore, our linear regression analysis indicated a positive correlation between *XBP1* and *ATF6* (Figure 2D; R^2^ = 0.4). Considering that ATF6 and the downstream transcription factor XBP1 restore homeostasis and induce apoptosis by regulating the Bcl-2 family proteins [59], we investigated the underlying relationship among them. As expected, we found a significant positive correlation between *XBP1* and *BCL-2* in schizophrenia (Figure 2E; R^2^ = 0.58). Interestingly, *XBP1* also displayed a significant positive association with *ERVW-1* (Figure 2F; R^2^ = 0.46). Taken together, *ATF6*, *BCL-2*, and *ERVW-1* displayed a significant correlation with *XBP1* in schizophrenia.

### 3.3. A Positive Correlation between ERVW-1 and ATF6-Mediated UPR and a Negative Correlation between GANAB and ERVW-1 in Recent-Onset Schizophrenia

**Figure 3:**
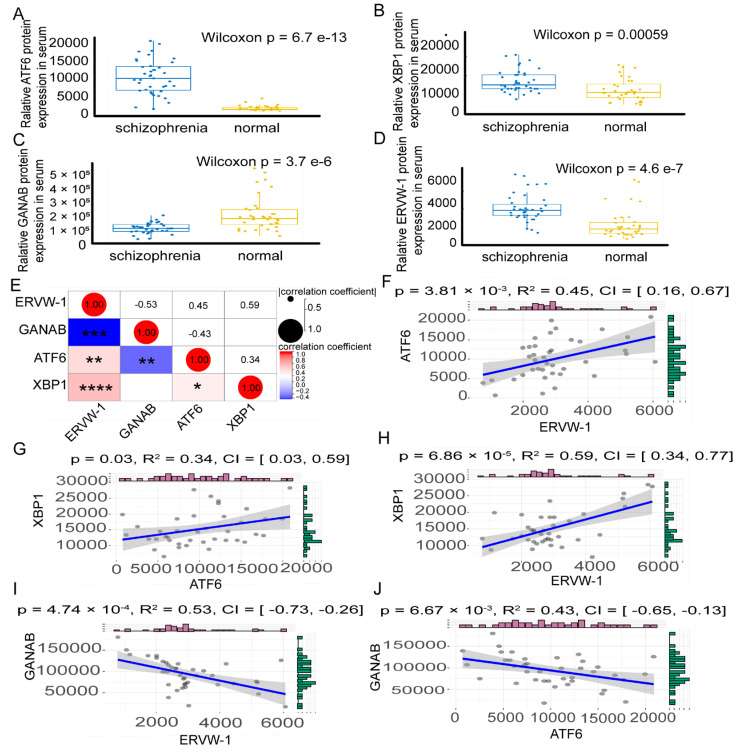
Upregulated expression of ATF6, XBP1 and ERVW-1 and downregulated expression of ATF6 in serum of subjects with recent-onset schizophrenia (n = 39) and healthy controls (n = 36) according to ELISA and multiple correlation among ERVW-1, GANAB, ATF6, and XBP1 in schizophrenia. (**A**) Wilcoxon signed rank test showed that the concentration of ATF6 was significantly higher in the serum of subjects with recent-onset schizophrenia than in normal controls (*p* < 0001, fold change = 16.05). (**B**) XBP1 was significantly increased (*p* < 0001, fold change = 1.04). (**C**) GANAB was significantly decreased (*p* < 0001, fold change = −1.30). (**D**) ERVW-1 was significantly increased (*p* < 0001, fold change = 2.48). (**E**) Multiple correlation analysis showed that ERVW-1 was significantly positively correlated with ATF6 and XBP1, that ATF6 was significantly positively correlated with XBP1, and that GANAB was negatively correlated with ERVW-1 and ATF6. (**F**) Linear regression correlation showed significant positive correlation between ERVW-1 and ATF6 (*p* < 0.05, R^2^ = 0.45). (**G**) ATF6 was significantly positively correlated with XBP1 (*p* < 0.05, R^2^ = 0.34). (**H**) ERVW-1 was significantly positively correlated with XBP1 (*p* < 0.0001, R^2^ = 0.59). (**I**) GANAB was significantly negatively correlated with ERVW-1 and GANAB (*p* < 0.001, R^2^ = 0.53). (**J**) GANAB was significantly negatively correlated with ATF6 (*p* < 0.05, R^2^ = 0.43). * *p* < 0.05, ** *p* < 0.01, *** *p* < 0.001, **** *p* < 0.001.

The enrichment analysis of GO biological processes has previously revealed the intricate connection between schizophrenia and the UPR. *ATF6B* and *ATF6*, which are activated by the inactive cyclic AMP-dependent transcription factor ATF6 during ER stress [13,60], were found to be the predominant DEGs and shared genes between schizophrenia DEGs and ER stress-related genes. Additionally, activated ATF6 translocates into the nucleus and induces the transcription of XBP1 mRNA [61]. The bioinformatic analysis of GSE21138 predicted a strong link between *ERVW-1* and *XBP1*. Consequently, we investigated whether ERVW-1 was correlated with ATF6-mediated unfolded protein genes, including *ATF6* and *XBP1*. First, we measured the protein expression of ATF6, XBP1, and ERVW-1 in serum samples from 39 patients and 36 healthy controls. There were no significant differences in age, level of education, body mass index (BMI), gender distribution, nor smoking status between schizophrenic patients and healthy controls (Table S1). Our results indicated that the serum ATF6 level was significantly higher in schizophrenic patients (Figure 3A). Similarly, the expression of XBP1, a downstream target of ATF6, was significantly elevated in schizophrenic patients (Figure 3B). Both the predictions of DEGs from GSE12649 and GSE21138 showed enrichment in N-glycan biosynthesis. Additionally, GANAB, the catalytic subunit of glucosidase II that specifically cleaves immature glycoproteins [17], contributes to N-glycan biosynthesis and the UPR [62]. We observed significantly lower serum concentration of GANAB in schizophrenic patients (Figure 3C). 

Consistent with our previous studies, the serum ERVW-1 level was significantly higher in schizophrenic patients (Figure 3D). Furthermore, we visualized the multiple correlations and regression analyses among ERVW-1, GANAB, ATF6, and XBP1 in the serum of individuals with recent-onset schizophrenia (n = 39) using a heatmap. We found a significant positive correlation between ERVW-1 and ATF6, as well as between ERVW-1 and XBP1. Conversely, GANAB showed a significant negative correlation with ERVW-1 and ATF6 (Figure 3E). Linear regression analysis revealed a positive correlation between ERVW-1 and ATF6 in schizophrenia (Figure 3F; R^2^ = 0.45), showing no difference from the results of the heatmap. ATF6 displayed a positive correlation with XBP1 (Figure 3G; R^2^ = 0.34). Consistently with our bioinformatic analysis results, there was a positive correlation between ERVW-1 and XBP1 (Figure 3H; R^2^ = 0.59). We also found a significant negative correlation between ERVW-1 and GANAB (Figure 3I; R^2^ = 0.53). Moreover, GANAB exhibited a negative correlation with ATF6 (Figure 3J; R^2^ = 0.43). In summary, there was aberrant expression of ATF6, XBP1, and GANAB in the serum of schizophrenic patients, and these proteins displayed significant correlations with ERVW-1 in schizophrenia.

### 3.4. ERVW-1 Increased ATF6 and XBP1 Expression and Enhanced Splicing Level of XBP1 in SH-SY5Y Cells

**Figure 4:**
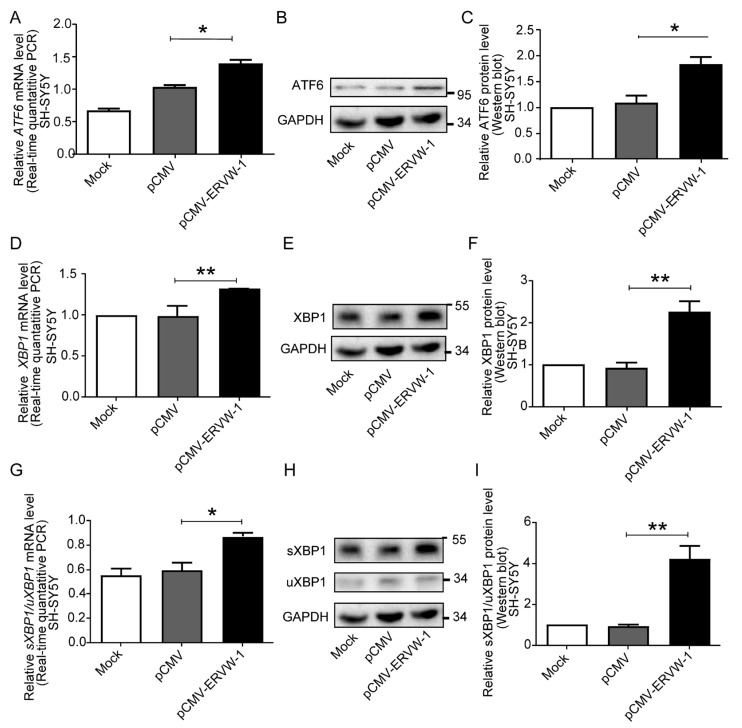
ERVW-1 could upregulate the expression of XBP1 and ATF6 and increase the splicing level of XBP1 in human neuroblastoma SH-SY5Y cells. (**A**) The relative mRNA level of *ATF6* was significantly increased by ERVW-1 (*p*, F, df, and n < 0.05, 2.144, 4, and 3). (**B**,**C**) The relative protein level of ATF6 was significantly increased by ERVW-1 (*p*, F, df, and n < 0.05, 1.082, 4, and 3). (**D**) The relative mRNA level of *XBP1* was significantly increased by ERVW-1 (*p*, F, df, and n < 0.01, 1510, 4, and 3). (**E**,**F**) The relative protein level of XBP1 was significantly increased by ERVW-1 (*p*, F, df, and n < 0.01, 4.291, 4, and 3). (**G**) The transcriptional ratio of *sXBP1/uXBP1* was significantly increased by ERVW-1 (*p*, F, df, and n < 0.05, 4.537, 4, and 3). (**H**,**I**) The translational ratio of sXBP1/uXBP1 was significantly increased by ERVW-1 (*p*, F, df, and n < 0.01, 53.49, 4, and 3). Statistical analysis: one-way ANOVA (* *p* < 0.05 and ** *p* < 0.01). All the experiments were repeated three times.

Our correlation analysis results revealed a positive correlation between ERVW-1 and ATF6 in patients with recent-onset schizophrenia. Therefore, we further investigated the relationship between ERVW-1 and ATF6 using the human neuroblastoma cell line SH-SY5Y. We successfully transfected *ERVW-1* into SH-SY5Y cells, as evidenced by increased ERVW-1 mRNA and protein levels (Figure S2A–C). Quantitative PCR results demonstrated that ERVW-1 increased the mRNA expression level of *ATF6* (Figure 4A). Subsequent Western blot experiments confirmed the alteration in ATF6 protein levels (Figure 4B,C). Given the positive correlation between ERVW-1 and XBP1, we conducted further studies on their relationship. We found that ERVW-1 significantly increased the expression of XBP1 at both mRNA (Figure 4D) and protein levels (Figure 4E,F). Interestingly, previous studies have reported that ATF6 induces *XBP1* mRNA [61], which aligns with our findings of a positive correlation between *ATF6* and *XBP1* in the bioinformatic analysis and correlation analysis in schizophrenia. Additionally, the spliced form of *XBP1* is an active transcription factor that plays a crucial role in the UPR. In ER stress, unspliced *XBP1*(*uXBP1*) is spliced and converted into the mature form encoding sXBP1 [63]. In this study, we observed a significantly higher ratio of *sXBP1/uXBP1* mRNA compared with the control group (Figure 4G). Similarly, the ratio of sXBP1/uXBP1 protein levels was increased compared with the control (Figure 4H,I). Our findings suggest that ERVW-1 upregulates ATF6 and XBP1 and also enhances the splicing level of XBP1.

### 3.5. ATF6 Was Involved in ERVW-1-Activated XBP1 Splicing in SH-SY5Y Cells

**Figure 5:**
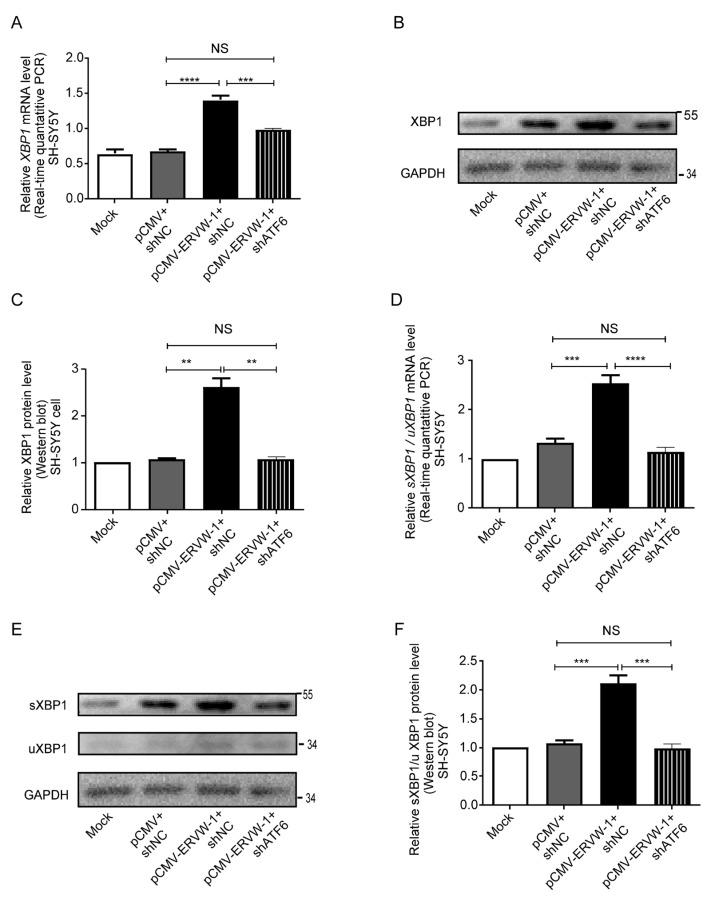
ATF6 mediated the upregulation and splicing level of XBP1 induced by ERVW-1 in SH-SY5Y cells. (**A**) qPCR showed that the transcriptional expression of *XBP1* induced by ERVW-1 was reversed by knocking down ATF6 in SH-SY5Y cells (*p*, F, df, and n < 0.001, 90.4, 2, and 3). (**B**,**C**) Western blot showed that the translational expression of XBP1 induced by ERVW-1 was reversed by knocking down ATF6 in SH-SY5Y cells (*p*, F, df, and n < 0.01, 64.96, 2, and 3). (**D**) qPCR showed that the transcriptional expressional ratio of *sXBP1/uXBP1* increased by ERVW-1 was reversed by knocking down ATF6 in SH-SY5Y cells (*p*, F, df, and n < 0.001, 42.44, 2, and 3). (**E**,**F**) Western blot showed that the translational expressional ratio of sXBP1/uXBP1 increased by ERVW-1 was reversed by knocking down ATF6 in SH-SY5Y cells (*p*, F, df, and n < 0.001, 39.44, 2, and 3). Statistical analysis: one-way ANOVA (** *p* <0.01, *** *p* < 0.001, and **** *p* < 0.0001). All the experiments were repeated three times.

ATF6 plays a role in maintaining ER homeostasis by inducing ER stress response elements, including XBP1 [64]. To investigate whether ATF6 mediates the regulation of ERVW-1-induced XBP1 splicing, we knocked down ATF6 expression and co-transfected it with *ERVW-1* in SH-SY5Y cells (Figure S2D–F). Silencing ATF6 substantially decreased the ERVW-1-induced mRNA expression of XBP1 in SH-SY5Y cells (Figure 5A). Consistently, the results of Western blotting showed that the downregulation of ATF6 could re verse the increase in XBP1 protein expression induced by ERVW-1 in SH-SY5Y cells (Figure 5B,C). These results suggest that *ERVW-1* increases the expression of XBP1 by upregulating *ATF6* expression. Additionally, ATF6 knockdown reduced the *ERVW-1*-induced splicing of XBP1 at both the transcriptional (Figure 5D) and protein levels (Figure 5E,F). In summary, ATF6 participated in the splicing of XBP1 regulated by ERVW-1.

### 3.6. ERVW-1 Mediated ER Stress with Inhibition of GANAB

**Figure 6:**
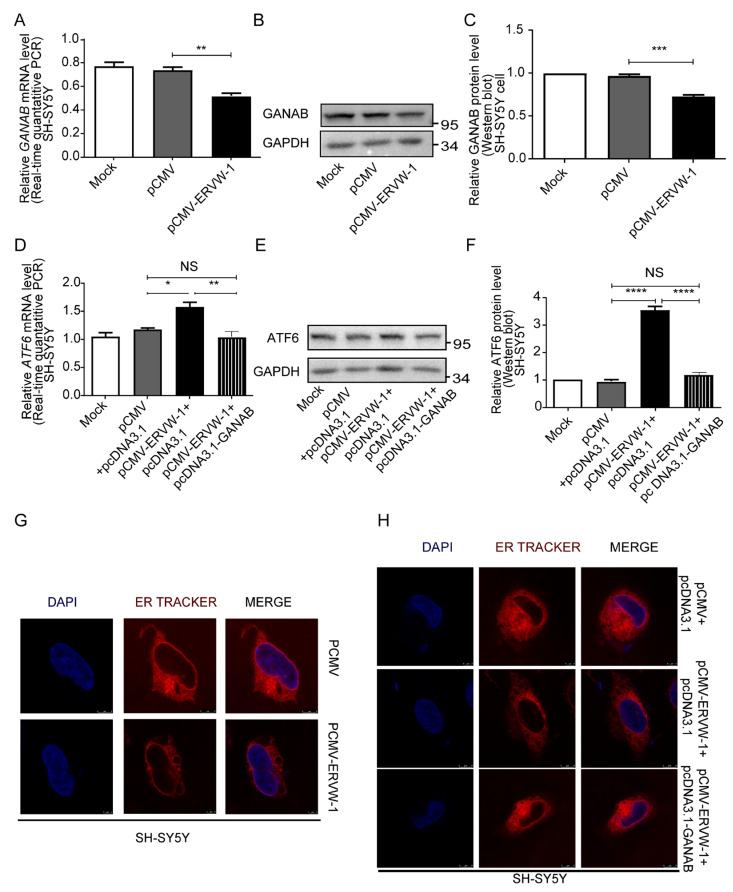
ERVW-1 upregulated ATF6 and induced ER vacuolization by inhibiting GANAB. (**A**) The mRNA level of *GANAB* was downregulated by ERVW-1 (*p*, F, df, and n < 0.01, 1.769, 4, and 3). (**B**,**C**) The protein level of GANAB was downregulated by ERVW-1 (*p*, F, df, and n < 0.001, 1.741, 4, and 3). (**D**) qPCR showed that the transcriptional expression of *ATF6* activated by ERVW-1 was reversed by increasing GANAB in SH-SY5Y cells (*p*, F, df, and n < 0.01 12.7, 2, and 3). (**E**,**F**) Western blot showed that the translational expression of ATF6 increased by ERVW-1 was reversed by increasing GANAB in SH-SY5Y cells (*p*, F, df, and n < 0.0001, 204, 2, and 3). (**G**) ERVW-1 could induce ER vacuolization. SH-SY5Y cells were transfected with pCMV or pCMV-ERVW-1 (100 pfu/cell) for 48 h followed by staining with ER tracker (red) and DAPI (blue). Images were then captured using a confocal laser scanning microscope (LEICA TCS-SP8). (**H**) ERVW-1-induced ER vacuolization was rescued by increasing GANAB. SH-SY5Y cells were co-transfected with pcDNA3.1 and pCMV-ERVW-1 or pcDNA3.1-GANAB and pCMV-ERVW-1 (100 pfu/cell). Statistical analysis: one-way ANOVA (* *p* < 0.05, ** *p* < 0.01, *** *p* < 0.001 and **** *p* < 0.0001). All the experiments were repeated three times.

It is well known that GANAB plays a crucial role in the N-linked glycosylation of proteins and protein processing in the ER [17]. Importantly, our clinical research revealed an inverse correlation between GANAB and ERVW-1. Therefore, we aimed to investigate the underlying molecular mechanism between GANAB and ERVW-1. As expected, ERVW-1 significantly downregulated GANAB expression at both the transcriptional (Figure 6A) and translational levels (Figure 6B,C). The UPR in mammalian cells operates via three main ER transmembrane receptors, one of which is ATF6 [18]. Upon cleavage and translocation to the nucleus, ATF6 activates ER stress response elements [65]. To determine whether *ERVW-1* upregulates ATF6 by inhibiting GANAB, we successfully co-transfected GANAB and ERVW-1 in SH-SY5Y cells (Figure S3A–C). Real-time PCR and Western blot results showed that GANAB inhibited the upregulation of ATF6 induced by *ERVW-1* (Figure 6D–F). These findings collectively suggest that ERVW-1-induces ATF6-mediated UPR and ER stress depending on GANAB downregulation.

Under normal physiological conditions, the ER is a large and continuous membrane-bound organelle composed of functionally and structurally distinct domains, which maintain its characteristic smooth structure [66]. However, in ER stress, misfolded proteins accumulate and overwhelm the cell’s capacity to maintain the ER secretory pathway, resulting in cellular vacuolization [67]. To investigate whether ERVW-1 induces ER vacuolization, we transfected SH-SY5Y cells with either the pCMV vector or pCMV-*ERVW-1* and stained the ER with ER-Tracker Red (red fluorescence; middle panel). Interestingly, when cells were transfected with the pCMV vector, the ER exhibited an intact and smooth pattern, and the ER-tracker signal was diffusely detected with only a few punctate dots on the ER (Figure 6G, top panel). However, in the presence of ERVW-1, the number of punctate dots increased, and most of them appeared as cup-shaped and ring-shaped structures, indicating ER vacuolization (Figure 6G, bottom panel). These changes in ER morphology provide further evidence of the role of ERVW-1 in activating the UPR and ER stress. To examine whether GANAB is involved in ER stress induced by ERVW-1, we co-transfected SH-SY5Y cells with pcDNA3.1-GANAB and pCMV-ERVW-1. We found that ERVW-1 indeed induced ER vacuolization (Figure 6H, middle panel). However, GANAB reduced the number and size of vacuoles in the ER, causing them to transition from circular structures to point-like structures. These results indicate that GANAB could alleviate ER vacuolization caused by ERVW-1 (Figure 6H, bottom panel). These changes in ER morphology provide further support for the notion that ERVW-1 mediates ER stress and the UPR with the inhibition of GANAB.

## 4. Discussion

ERVW-1 has been found in the CSFs and serum of individuals with recent-onset schizophrenia [37,38]. Furthermore, the transcriptional level of *ERVW-1* is significantly upregulated in brain mRNA-seq data [58]. Our previous studies have demonstrated that ERVW-1 is a possible etiology of schizophrenia [21,36,39,40,41,42,43,44,51,52,53,54,55,56,57]. Moreover, our in-depth studies have shown that ERVW-1 contributes to schizophrenia in several different ways. Firstly, ERVW-1 regulates immune-related pathways, including the activation of CRP via TLR3 [51] and the induction of TNF-α production via TLR4 [57]. Additionally, ERVW-1 increases nitric oxide (NO) synthase, induces microglial cell migration [54], and activates robust cytotoxic T-lymphocyte (CTL) responses [55]. Recently, our lab has reported that ERVW-1 also induces innate immunity and apoptosis via the linc01930/cGAS axis [44]. Secondly, ERVW-1 affects ion channels. It can regulate calcium influx by activating TRPC3 [56], thereby triggering Ca^2+^-activated K^+^ type 2 channels (SK2, also called KCNN2) and SK3 [56]. Furthermore, ERVW-1 can activate sodium channels [42,52]. Thirdly, the aberrant expression of ERVW-1 activates CPEB1, leading to dysregulated mitochondrial dynamics and abnormal energy metabolism [40]. Further studies indicate that ERVW-1 induces unnatural dopaminergic neuron proteins, including BDNF, CREB and GSK3β [39,42,53]. In this paper, we found that ATF6 and XBP1 could be potential serum biomarkers of schizophrenia. Further studies indicated that ERVW-1 induced ER stress and ER vacuolization and that GANAB participated in those processes caused by ERVW-1, showing the potential mechanism of aberrant protein homeostasis in the ER in the pathogenesis of schizophrenia.

Schizophrenia is estimated to have a heritable component, although molecular genetic research has not yet provided conclusive evidence. Microarray analysis is an important tool for investigating the interaction of schizophrenia-related genes and plays a significant role in identifying biomarkers of and drug targets for schizophrenia [68]. Post-mortem brain microarrays [68], such as GSE12649 [45] and GSE21138 [47], are important classical microarrays used in schizophrenia studies. Functional changes in the brain have been observed in schizophrenic patients, and the connectivity of the prefrontal cortex with other brain regions is crucial for cognitive function [3,69]. A better understanding of the molecular mechanisms underlying the prefrontal cortex could have important implications for schizophrenia therapeutics. Iwamoto, K., et al. demonstrated a global downregulation of mitochondrial genes in both bipolar disorder and schizophrenia using GSE12649 [45]. However, our bioinformatic analysis of GSE12649 revealed a link between schizophrenia and ER stress. Additionally, a pathway analysis identified three schizophrenia risk pathways, including EIF2-, IGF-1-, and 14-3-3-mediated signaling pathways [70]. Gene-based and pathway-based meta-analyses of GSE12649 suggested that multiple pathways, including bile acid metabolism, complement, p53 pathway, apoptosis, and inflammatory response, may be associated with schizophrenia [71]. However, meta-analysis and pathway analysis require normalization to integrate different schizophrenia datasets, which may result in the loss of potential pathways and biological functions, such as ER stress [70,71]. Here, we discovered an association between ER stress and schizophrenia. Subsequently, our analysis revealed decreased expression of *ATF6* and *ATF6B* at the transcriptional level. The ATF6 precursor is converted into active transcription factors, ATF6 and ATF6B, which are involved in the UPR [13,60]. The transcriptional repression of ATF6B to ATF6 and its proteolytic cleavage depend on the N-linked glycosylation sites of ATF6B [72]. The glycosylation of ATF6 serves as a sensor of ER homeostasis [73], and aberrant glycosylation can lead to ER stress [74]. Our pathway enrichment analysis of schizophrenia using GSE12649 found that the DEGs were enriched in glycosylation-related pathways, including N-glycan biosynthesis, gluconeogenesis, and various types of N-glycan biosynthesis.

GSE21138 is another large-scale DNA microarray dataset of mRNA obtained from the prefrontal cortices of individuals with schizophrenia and healthy controls. A clustering analysis of GSE21138 showed that DEGs in schizophrenia are enriched in glycosylation, protein localization, and protein transport processes [47]. These biological processes are colocalized with ER stress in subcellular compartments [75,76,77], indirectly supporting our bioinformatic prediction of a link between ER stress and schizophrenia. Additionally, GSE21138 includes the probe for ERVW-1, which is not present in GSE12649 [45,47]. The bioinformatic analysis of GSE21138 indeed showed a significant positive correlation between *ATF6* and *XBP1*. Interestingly, in GSE21138, ERVW-1 exhibited a significant positive correlation with XBP1 at the transcriptional level in the prefrontal cortex of individuals with schizophrenia, suggesting a connection among *ERVW-1*, *ATF6*, and *XBP1*. In summary, the bioinformatic analysis provided evidence that *ATF6* could serve as a biomarker for the early detection of schizophrenia.

The identification of effective biomarkers is crucial for the diagnosis and prediction of schizophrenia. Currently, there is no objective biomarker available for detecting schizophrenia [78]. Therefore, there is an urgent need to discover reliable biomarkers, especially serum biomarkers, for schizophrenia. Various reliable biomarkers for schizophrenia have been reported, including inflammation biomarkers, neurotrophic biomarkers, neurotransmitters, and major schizophrenia-related genes [79]. Additionally, our previous research suggested that CRP [80], IL10 [81], and BDNF [82], which are regulated by ERVW-1, can be used as serum biomarkers of schizophrenia. Furthermore, ATF6 is known as a brain biomarker for various nervous system diseases, including amyotrophic lateral sclerosis (ALS), Alzheimer’s disease (AD) [83], and neonatal hypoxic–ischemic encephalopathy [84], but whether ATF6 can be used as a biomarker of schizophrenia has not been reported. In this study, we reported an increase in ATF6 expression in schizophrenia and its correlation with ERVW-1. Moreover, XBP1, a downstream target gene of ATF6, is a brain biomarker of ALS and AD [83]. Our clinical experiment indicated that XBP1 expression was higher in schizophrenic patients than in healthy controls and displayed a positive correlation with ERVW-1. Interestingly, we also identified abnormal expression of GANAB, a catalytic subunit of glucosidase II involved in glycosylation, in individuals with schizophrenia. Although GANAB is a biomarker of neurological diseases, including MS [85] and traumatic brain injury [86], its potential as a biomarker of schizophrenia has not been reported. To the best of our knowledge, there are no studies reporting the effects of these biomarkers on antipsychotic drug treatment or behavioral therapy for schizophrenia. Therefore, we propose that ATF6, XBP1, and GANAB might serve as potential serum biomarkers of schizophrenia.

The upregulation of ATF6 and XBP1 may lead to ER stress, which is caused by protein misfolding and aggregation, and plays a critical role in neurodegeneration [87,88]. The activation of PERK signaling has been reported in post-mortem brain tissue from PD patients [89], and increased *XBP1* splicing has been observed in AD patients [90]. Furthermore, ATF6 has been found to be closely associated with neurodegenerative diseases. The abnormal expression of ATF6 is implicated in diseases such as ALS [91] and Huntington’s disease [92]. Although the precise mechanisms are not fully understood, there is substantial evidence demonstrating that factors such as Ca^2+^ channels [93], cochaperone SIL1 [94], and the UPR core gene *IRE1* [95] can contribute to the accumulation of misfolded proteins, leading to ER stress. Despite the strong connection between ER stress or UPR and various neurodegenerative diseases, there is a significant lack of studies investigating ER stress in schizophrenia. Intriguingly, our previous findings demonstrate that ERVW-1, a risk factor for schizophrenia, also regulates calcium potential [56,93] in schizophrenia. Decreased neuroplasticity [96], severe neuroinflammation [97], and extensive neuronal cell death [98] have been implicated in schizophrenia, and these biological processes are closely related to ER stress [4,6,7]. Furthermore, retroviruses can induce ER stress response [99]. Specifically, ERVW-1 can induce ER stress via TNF-α [100] and upregulate ER chaperones, including CHOP, Bip, PERK ERp75, and OASIS, in astrocytes [101]. Therefore, we investigated the relationship among ERVW-1, ATF6, and XBP1 with cytological experiments using the neuroblastoma cell line SH-SY5Y. We confirmed that ATF6 mediated UPR alterations in the presence of ERVW-1. The cytological results showed that ATF6 and XBP1 were upregulated at both the transcriptional and translational levels upon the overexpression of ERVW-1. Profound studies suggested that ERVW-1 increases the expression and splicing of XBP1 by upregulating ATF6. Our results suggest that ERVW-1 might participate in schizophrenia by upregulating ATF6.

Both the UPR and ER stress are closely associated with glycosylation, and the dysregulation of glycosylation may cause the UPR [18,19]. The recognition and transfer of misfolded glycoproteins rely on protein N-glycosylation in ER-associated degradation [102]. ER stress can be induced by perturbed protein N-glycosylation [103]. XBP1 is involved in N-glycoprotein biosynthesis, and there is an important link between UPR and N-glycan [104]. The UPR core gene sXBP1 combines with protein N-glycosylation to protect cells [105]. Interestingly, the perturbation of glycation results in ER stress, which contributes to neurodegenerative disorders [106], including AD [107] and PD [108]. The glycosylation modification of cyclic adenosine monophosphate (cAMP) response element-binding protein is involved in the development of neurodegenerative diseases [109]. Glycosylation is a common mechanism in neurodegenerative diseases [110]. GANAB is a catalytic subunit of glucosidase II [17]. GANAB activates ATF6 and later leads to MS [111]. However, there are no available studies on the mechanism of GANAB in schizophrenia. In this paper, we investigated the potential link between GANAB and schizophrenia. First of all, both GANAB and ERVW-1 showed a significant correlation with ATF6 and XBP1 in the serum of schizophrenic patients. Moreover, we found that ERVW-1 could decrease GANAB transcript and protein levels and that *GANAB* could inhibit the increase in ATF6 expression induced by ERVW-1. In conclusion, *GANAB* participates in the *ERVW-1*-induced ATF6-mediated UPR pathway, leading to ER stress and contributing to the pathogenesis of schizophrenia.

ER stress impacts the morphology of the ER [112,113]. The accumulation of misfolded protein overwhelms the ability to maintain ER secretion, resulting in ER vacuolization [67]. For instance, XBP1 can induce a wide spectrum of secretory pathway genes and physically expand the ER [114]. XBP1, which links the UPR to phospholipid biosynthesis, causes increased the surface area and volume of the rough ER [115]. Subsequently, perturbed lipid composition and localization can induce ER expansion [116] and compromise ER membrane integrity [117], leading to ER vacuolization. The aberrant expression of ER stress-related genes, including hsp70 heat shock family member Bip [118] and Rab7a [119], can contribute to ER vacuolization. ER vacuolization is associated with multiple neurological disorders [120], especially single-nucleotide polymorphism of the receptor *CALCOCO1*, which increases the risk of tardive dyskinesia in schizophrenia [121]. Furthermore, schizophrenia microglia cells show significant ER vacuolization, including increases in ER volume and vacuole number [122]. However, there are no studies on ER vacuolization related to ERVW-1, an important risk factor for schizophrenia. Intriguingly, our confocal experiments showed that ERVW-1 significantly increased ER vacuolization and that ERVW-1-induced enhanced ER vacuolization was suppressed by GANAB, suggesting that ERVW-1 leads to ER vacuolization and activates ER stress. Furthermore, this process was mediated by decreasing the expression of GANAB, resulting in a change in morphology and dysregulated homeostasis in the ER. Consequently, ER vacuolization may play a role in the etiology of schizophrenia.

## 5. Conclusions

**Figure 7:**
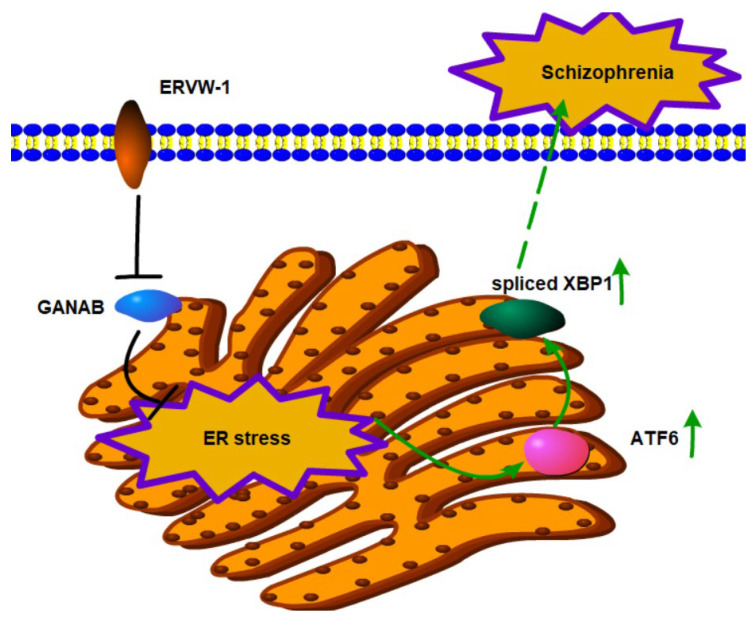
ERVW-1 activates ER vacuolization and ER stress via ATF6/spliced XBP1 signal by inhibiting GANAB in the etiology of schizophrenia. ERVW-1 can inhibit the expression of GANAB. Inhibited GANAB participates in ER vacuolization and ER stress induced by ERVW-1. After ER stress, ATF6-mediated UPR pathway genes, including ATF6 and spliced XBP1, are increased, which affects homeostasis in the ER, contributing to the development of schizophrenia.

Our bioinformatic analysis of mRNA samples from the prefrontal cortex of schizophrenic patients and healthy controls indicated an increased level of *ATF6* in schizophrenia. There is a positive correlation between *ATF6* and its downstream target gene *XBP1*. Additionally, a positive correlation between *ERVW-1* and *XBP1* was observed. Furthermore, enrichment analysis showed a relationship among UPR, N-glycan biosynthesis, and schizophrenia. Similarly, our clinical data also suggest increased serum levels of ATF6, XBP1, and ERVW-1 in schizophrenic patients compared with healthy controls, and a positive correlation was found among ATF6, XBP1, and ERVW-1. In vitro studies demonstrated that ERVW-1 could upregulate ATF6 and increase XBP1 expression and splicing. Moreover, in vitro studies revealed that ERVW-1 could inhibit GANAB expression and that GANAB participated in the increase in ATF6 induced by ERVW-1. Interestingly, further studies indicated that ERVW-1 induced ER stress, and this effect was mediated by GANAB. These findings may provide a new molecular mechanism associated with schizophrenia, suggesting that ERVW-1 induces ER stress by downregulating GANAB, contributing to the pathogenesis of schizophrenia (Figure 7).

## Data Availability

The data that support the findings of this study are available from Gene Expression Omnibus at Home—GEO—NCBI (nih.gov) (accessed on 29 August 2022), reference numbers GSE12649 and GSE21138. These data were derived from the following resources available in the public domain: GSE12649, GEO Accession viewer (nih.gov); GSE21138, GEO Accession viewer (nih.gov).

## Supplementary Materials

The following supporting information can be downloaded at: https://www.mdpi.com/article/10.3390/v15061298/s1, Figure S1: 28 common genes between DEGs from GSE12649 and ER stress gene sets, DEGs from GSE12649 were functionally enriched in ER stress-related cellular components and molecular functions, Table S1: Univariate and multivariate analyses of risk factors for schizophrenia, Figure S2: ERVW-1 upregulated, and ATF6 silencing could rescue the increased expression of ATF6 induced by ERVW-1 in SH-SY5Y cells, Table S2: List of genes and primer sequences used for qPCR, Figure S3: Overexpression of GANAB could reverse the decrease of GANAB induced by ERVW-1 in SH-SY5Y cells.
