# Supplementary material for: ERVW-1 Activates ATF6-Mediated Unfolded Protein Response by Decreasing GANAB in Recent-Onset Schizophrenia
Source: Viruses. 2023 May 31;15(6):1298. doi: 10.3390/v15061298 (PMC10304270; doi:10.3390/v15061298)
Supplement: Supplementary file 1 [file viruses-15-01298-s001.zip › viruses-2397883-supplementary.pdf]

Supplementary Materials:

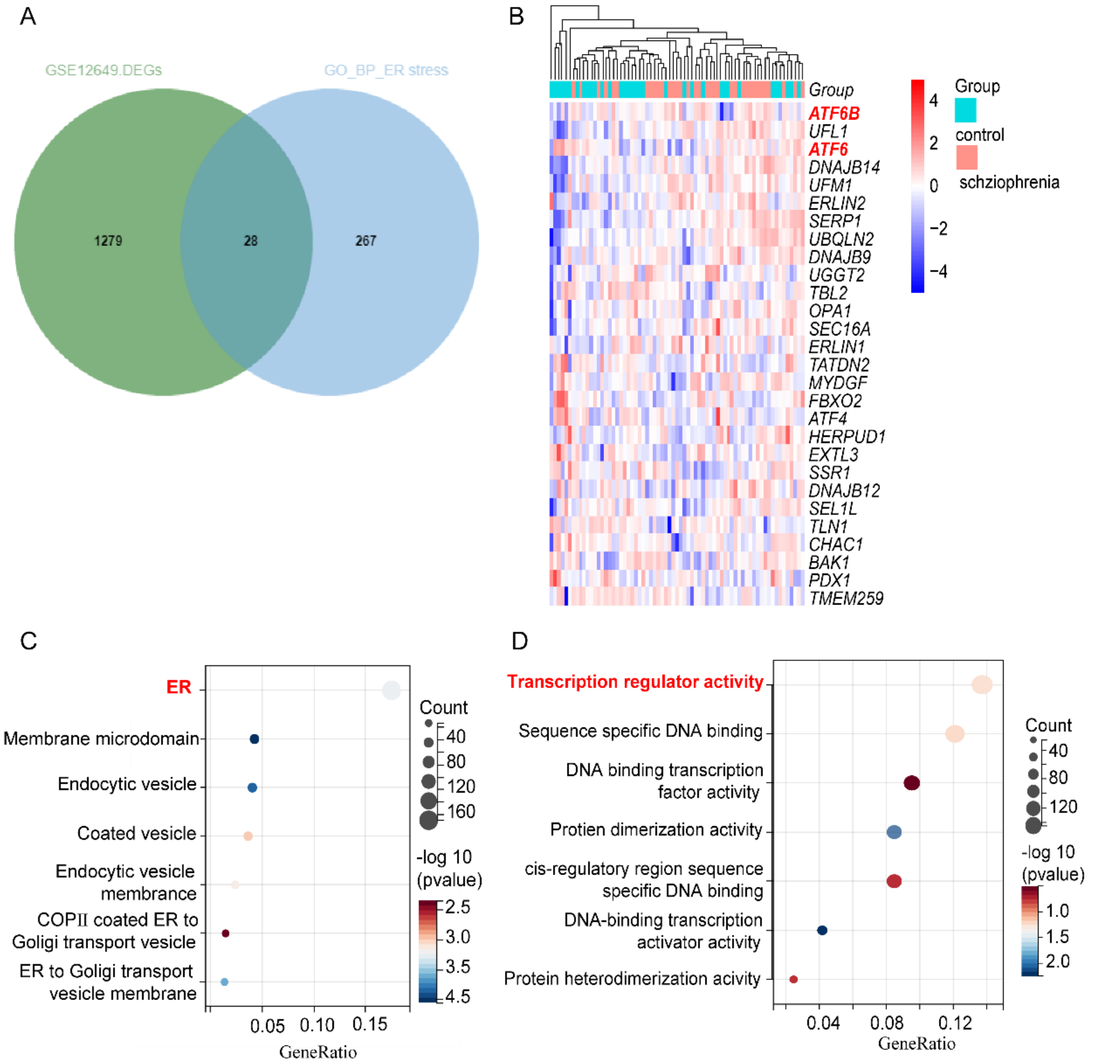

Figure S1. 28 common genes of DEGs from GSE12649 and ER stress gene sets, DEGs from GSE12649 were functional enriched in ER stress related cellular component and molecular function (**A**) Venn plot showed 28 common genes between schizophrenia and ER stress. (**B**) Heatmap showed the expression of 28 common genes between ER stress and DEGs in individuals with schizophrenia and normal controls. (**C**) GO cellular component enrichment analysis showed DEGs were enriched in ER. (**D**) GO molecular function enrichment analysis showed that DEGs were enriched in transcription regulation activity.

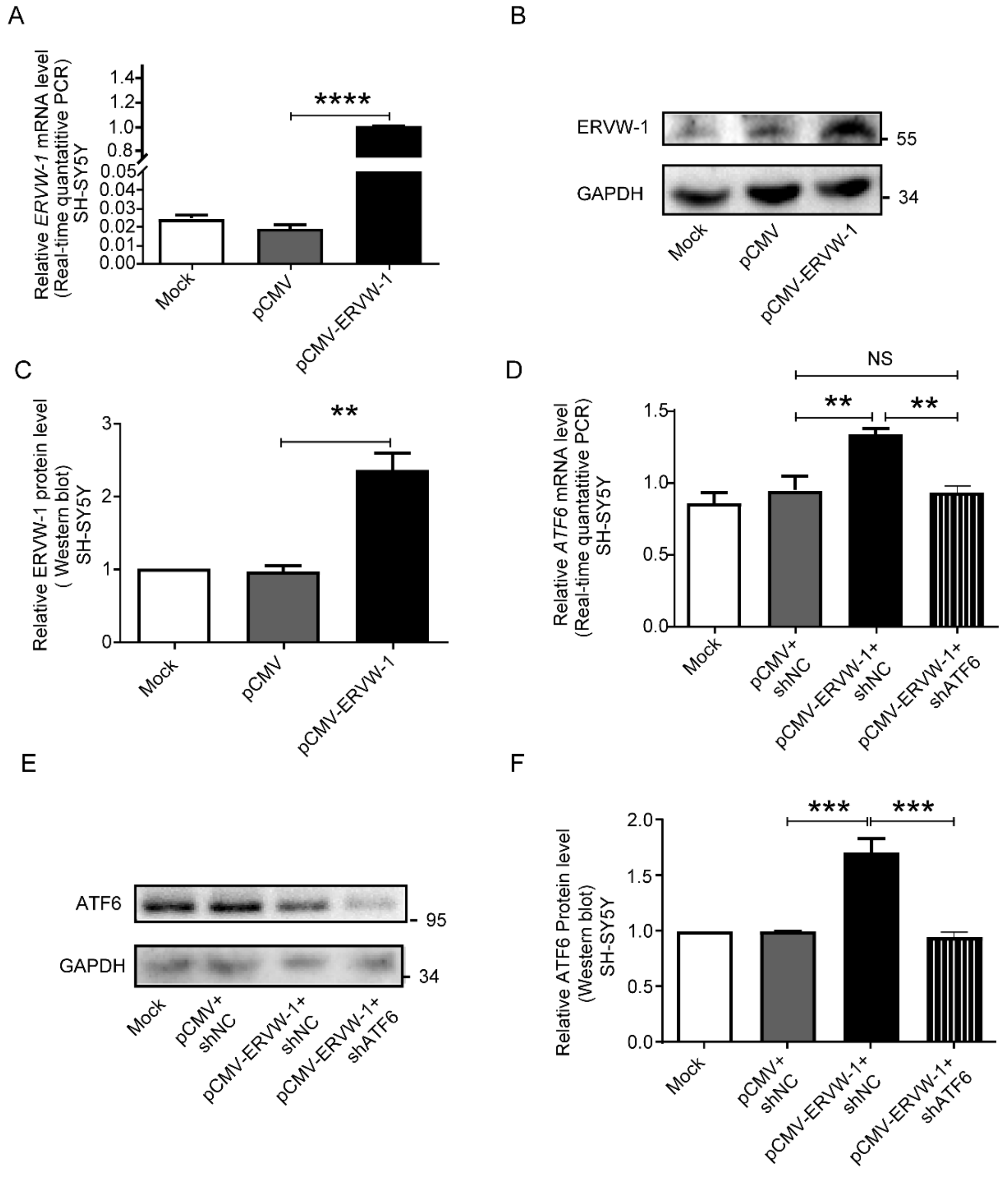

Figure S2. ERVW-1 upregulated and ATF6 silencing could rescue the increased expression of ATF6 induced by ERVW-1 in SH-SY5Y cells. (**A**) The transcriptional expression of *ERVW-1* was significantly overexpressed after transfection with ERVW-1 in SH-SY5Y cells using qPCR (p, F, df, n, < 0.0001, 1.848, 4, 3). (**B**, **C**) Western blot showed that ERVW-1 was significantly overexpressed after transfected with ERVW-1 in SH-SY5Y cells (p, F, df, n, <0.01, 2.172, 4, 3). (**D**) The increased transcriptional expression of *ATF*6 induced by ERVW-1 was reversed after co-transfection with ERVW-1 and shATF6 in SH-SY5Y cells (p, F, df, n, < 0.01, 9.035, 2, 3). (**E**, **F**) Western blot showed that increased protein levels of ATF6 was reversed after co-transfection with ERVW-1 and shATF6 in SH-SY5Y cells (p, F, df, n, < 0.001, 2990, 2, 3). Statistical analysis: one-way ANOVA (*p < 0.05, **p <0.01, ***p < 0.001, **** p < 0.0001). All the experiments were repeated three times.

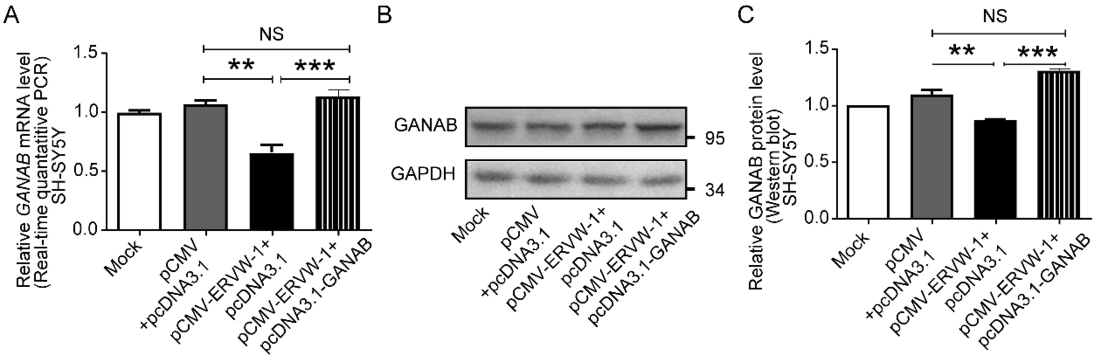

Figure S3. Overexpression of *GANAB* could reverse the decrease of GANAB induced by ERVW-1 in SH-SY5Y cells. (**A**) The inhibited transcriptional expression of GANAB by ERVW-1 was reversed after co-transfection with ERVW-1 and GANAB in SH-SY5Y cells using qPCR (p, F, df, n, < 0.001, 25.43, 2, 3). (**B, C**) The western blot results of GANAB after co-transfection with *ERVW-1* and *GANAB* in SH-SY5Y cells (p, F, df, n, < 0.001, 70.15, 2, 3). Statistical analysis: one-way ANOVA (*$p < 0.05$, **$p < 0.01$, ***$p < 0.001$). All the experiments were repeated three times.

Table S1. Univariate and multivariate analysis of risk factors for schizophrenia

	**Onset Schizophrenia (N = 39)**		**Healthy controls (N = 36)**		**Analysis**
	Median	Range	Median	Range	*P*
Age (years)	45	20-66	43.5	23-65	0.820
Education (years)	12	3-16	12	3–16	0.876
BMI (body mass index)	21.2	16.9-26.4	22.6	15.1-27.5	0.149
	N	%	N	%	*P*
Gender					
Male	18	46	17	47	0.926
Female	21	54	19	53	
Smoking status					
Yes	15	38	14	39	0.97
No	24	62	22	61	

Table S2. List of genes and primer sequences used for qPCR

**Target gene**	**PCR product size**	**Oligo sequence (5' > 3')**
ATF6 (NM_001410890.1)	93bp	F-CAGGAACTCAGGGAGTGAGC
		R-AATGTGTCTCCCCTTCTGCG
ERVWE1 (NM_014590.4)	78bp	F-CCAATGCATCAGGTGGGTAAC
		R-GAGGTACCACAGACAAAAAATATTCCT
GANAB(NM_001278192.2)	181bp	F-GCGTAGGAGGCGGCTTTC
		R-GGCCCTCTGATGCTCAAACT
XBP1s(NM_001393999.1)	130bp	F- GCTGAGTCCGCAGCAGGT
		R- CTGGGTCCAAGTTGTCCAGAAT
XBP1u(NM_001394000.1)	139bp	F-CAGACTACGTGCACCTCTGC
		R- CTGGGTCCAAGTTGTCCAGAAT
